# Menopausal Hormone Therapy in Clinically Vulnerable Women: A Narrative Review of Guidelines and Real-World Evidence

**DOI:** 10.3390/medicina62040712

**Published:** 2026-04-08

**Authors:** Vesselina Yanachkova, Hristina Lebanova, Svetoslav Stoev

**Affiliations:** 1 Research Institute, Medical University-Pleven, 5800 Pleven, Bulgaria; 2 Department of Endocrinology, Specialized Hospital for Active Treatment of Obstetrics and Gynecology “Dr Shterev”, 1330 Sofia, Bulgaria; 3 Department of Pharmaceutical Sciences and Social Pharmacy, Faculty of Pharmacy, Medical University-Pleven, 5800 Pleven, Bulgaria; hristina.lebanova@mu-pleven.bg

**Keywords:** menopause, menopausal hormone therapy, vulnerable populations, multimorbidity, frailty, polypharmacy, clinical guidelines, EMAS, NAMS, NICE, ACOG

## Abstract

*Background and Objectives:* Menopausal hormone therapy (MHT) is the most efficacious treatment for vasomotor symptoms and genitourinary conditions associated with menopause. Modern menopause care increasingly encompasses women with multimorbidity, renal or hepatic impairment, previous malignancies or thromboembolic disorders, advanced age, and polypharmacy—groups frequently underrepresented in randomized clinical trials. This evidence gap prompts significant inquiries about the relevance of trial-based recommendations to actual clinical practice. *Materials and Methods:* This narrative review offers a concentrated assessment of prominent worldwide clinical guidelines regarding menopausal hormone therapy through thematic synthesis. We examined position statements from the North American Menopause Society (NAMS), the European Menopause and Andropause Society (EMAS), NICE clinical guidelines, the ACOG Practice Bulletin on menopausal symptom management, the Endocrine Society clinical practice guideline, and pertinent UK guidance from RCOG, BMS, and BGCS. Data from systematic reviews, meta-analyses, and extensive observational studies were analyzed to contextualize guideline recommendations for populations often underrepresented in clinical trials, including women aged ≥65 years and individuals with multimorbidity or polypharmacy. *Results:* Only the NICE and EMAS recommendations expressly acknowledge clinical vulnerability or complexity (multimorbidity, frailty, and cancer survivorship) as foundational principles. NAMS and ACOG delineate risk categories but fail to offer a cohesive taxonomy of vulnerability. Polypharmacy and drug–drug interactions are inconsistently addressed across guidelines, and there is a deficiency of standardized prescribing algorithms. While routine safety monitoring is universally advocated, the intervals for follow-up and methods for risk categorization differ. Observational evidence consistently indicates route-dependent variations in cardiovascular and thromboembolic risk, with transdermal estrogen linked to a more advantageous safety profile in higher-risk individuals. *Conclusions:* Present menopausal therapy guidelines are methodologically sound; however, they insufficiently address the complexities of multimorbidity, polypharmacy, and organ dysfunction. A systematic conceptual framework that incorporates areas of clinical vulnerability may facilitate personalized benefit–risk evaluation in practical applications. Future guideline revisions should enhance clarity by incorporating polypharmacy concerns, monitoring strategies, and systematic risk stratification methods for clinically complicated patients.

## 1. Introduction

Menopausal hormone therapy remains the gold standard for treating vasomotor symptoms and some urogenital menopausal symptoms; it has been shown to improve bone density and reduce the risk of osteoporotic fractures. Despite these advantages, there is still much clinical and scientific disagreement on the use of systemic menopausal hormone therapy in older women, particularly those over 65. Evidence from randomized controlled trials and subsequent meta-analyses indicates that the benefit–risk ratio of menopausal hormone therapy is significantly influenced by the age at which medication is commenced, the duration after menopause, and the specific risk profile of patients [1,2,3,4,5,6,7]. Current recommendations and guidelines for MHT in menopausal women converge on the principle known as the “timing hypothesis” [8]. This hypothesis posits that the benefit–risk ratio of systemic MHT is most advantageous for women under 60 years of age and/or when initiated within the first 10 years post-menopause. Initiating therapy at a later stage is associated with an elevated absolute risk of cardiovascular events, venous thromboembolism, and cognitive problems, necessitating heightened vigilance and more stringent individualization of the therapeutic strategy.

Women aged over 65 constitute a clinically diverse yet high-risk demographic, marked by a significant prevalence of cardiovascular disease, type 2 diabetes mellitus, dyslipidemia, obesity, and cognitive and emotional disorders [9,10,11,12]. These comorbidities significantly influence the occurrence of adverse events linked to systemic menopausal hormone therapy, such as venous thromboembolism, stroke, and coronary incidents, especially when treatment is commenced later post-menopause [1,2,3,4,5,6,13]. The conventional approach to discontinuing MHT after the age of 65 is gradually being reassessed. Recent observational studies indicate that the risk profile associated with continuing therapy beyond this age significantly differs based on the type of hormone, dosage, and method of administration. Despite the limitations of retrospective analyses, these data advocate for an alternative strategy wherein ongoing menopausal hormone therapy may be warranted in carefully chosen patients exhibiting chronic symptoms, dependent upon the administration of the minimal effective dosage and the implementation of regular clinical evaluations. Due to the negative benefit–risk ratio, it is still typically not advised for women over 65 to begin systemic MHT de novo.

Expert opinions state that MHT is not recommended for the primary or secondary prevention of cardiovascular disease. The initiation or continuation of therapy, particularly in vulnerable groups such as older, multimorbid women, must stem from an informed, collaborative decision-making process, supplemented by ongoing risk reassessment. The route of administration of MHT is also of particular significance. The oral administration of estrogen is noteworthy due to its substantial first-pass hepatic metabolism, resulting in the elevated production of coagulation factors and alterations in lipid and inflammatory profiles. These effects may be clinically significant in patients with elevated thrombotic or cardiovascular risk and could be exacerbated in the context of polypharmacy. While transdermal estrogen formulations are regarded as a safer option for certain individuals, evidence regarding their application in women with a significant drug burden is limited.

A significant, although frequently undervalued, factor among this cohort of women is the prevalence of polypharmacy. Polypharmacy, typically characterized by the concurrent administration of five or more drugs, is a recognized independent risk factor for adverse drug responses, drug interactions, functional deterioration, and heightened mortality in elderly individuals [5,6]. Observational pharmacoepidemiologic data indicate that women undergoing systemic menopausal hormone therapy while on polypharmacy may face an increased risk of interaction-related safety alerts compared to those utilizing fewer concurrent drugs. Nonetheless, these findings are to be regarded as connections rather than proof of direct causality. In clinical practice, women in this demographic frequently utilize antihypertensives, anticoagulants or antiplatelet drugs, lipid-lowering pharmaceuticals, antidiabetic agents, and psychiatric medications, resulting in complex pharmacokinetic and pharmacodynamic interactions. While the majority of existing meta-analyses and systematic reviews evaluate the risk of menopausal hormone therapy for specific outcomes, such as venous thromboembolism, stroke, or breast cancer, the influence of polypharmacy as a potential risk modifier is inadequately explored. Most clinical trials lack a comprehensive study of the quantity and nature of concurrent drugs, despite this characteristic mirroring the actual clinical setting for older women.

In patients with multimorbidity, the baseline risk of adverse events is increased, which complicates therapeutic decisions. The presence of established atherothrombotic cardiovascular disease, previous venous thromboembolism, or active oncological disease is usually a contraindication to the initiation of systemic MHT. In these cases, priority is given to the use of non-hormonal therapeutic approaches or topical estrogen preparations in the presence of genitourinary syndrome of menopause.

The guidelines for menopausal hormone therapy in postmenopausal women necessitate substantial revision, especially considering improved survival rates from chronic illnesses and the changing quality of life expectations among middle-aged and older women. A considerable number of postmenopausal women pursue assistance and initiate menopausal hormone therapy later in the postmenopausal transition, at which point they often exhibit established multimorbidity alongside associated polypharmacy, cardiovascular complications, altered metabolic profiles, and a history of oncological, gynecological, or iatrogenic interventions, including cancer treatment and risk-reducing surgeries. Clinical treatment and advice for postmenopausal women are perceived as increasingly complex, contrasting with previous, simplistic benefit–risk frameworks based on studies involving younger, healthier populations. Concurrently with these alterations in patient demographics, the analysis of evidence pertaining to menopausal hormone therapy has progressed. Recent guidelines, informed by extensive follow-up of large randomized trials and the aggregation of data from observational and meta-analytic studies, increasingly stress the importance of enhancing risk assessment by differentiating between baseline risk, treatment-related risk modification, and the influences of age, time since menopause, comorbidities, and hormone administration route [9,10,11,12]. This change has resulted in a transition from generalized avoidance of hormone therapy to a more personalized strategy grounded in benefit–risk assessment.

In this context, numerous prominent professional organizations—such as the North American Menopause Society, the European Menopause and Andropause Society, the NICE NG23 guidelines, and various UK collaborative guidelines on menopausal management post-gynecological cancer—have reiterated that menopausal hormone therapy is the most efficacious first-line treatment for troublesome vasomotor and genitourinary symptoms in suitably chosen women. This support is mostly contingent upon personalized risk–benefit discussions, collaborative decision-making, and ongoing reassessment, rather than rigid age thresholds or definitive time limits since menopause.

These guidelines consistently characterize MHT not as a standardized therapy but as a personalized intervention, with recommendations customized according to symptoms, baseline cardiovascular and thromboembolic risk, breast cancer risk, comorbidities, prior cancer history, and patient preferences. There is a growing focus on the route of administration, dosage determination, and monitoring techniques, especially for women with intricate medical backgrounds. The guideline authors recognize ongoing evidence deficiencies in key populations—specifically women aged ≥65 years, late initiators, and individuals with multimorbidity and polypharmacy—who are increasingly prevalent in real-world clinical practice yet remain underrepresented in randomized controlled trials. Women most prone to severe or persistent symptoms are frequently those underrepresented in studies: older women (>65 years), surgically menopausal following pelvic malignancy, with chronic kidney or liver disease, with a history of venous thromboembolism (VTE) or thrombophilia, or those on 5–10 chronic medications [13].

The primary inquiry of this article is thus straightforward: Do existing menopausal recommendations provide direction for the management of these women? Recent research and systematic reviews have enhanced the evidence about the safety and efficacy of menopausal hormone therapy and nonhormonal alternatives for elderly and clinically complex women. These statistics underscore the necessity of tailoring guideline recommendations for susceptible categories, including individuals with multimorbidity, organ damage, a history of malignancy, or polypharmacy. Recent research highlights critical domains such as cardiovascular and thrombotic risk, cognitive health, interaction burden, and advancements in non-hormonal therapy.

Collectively, these advancements highlight the transition from a primarily age-based and risk-averse framework to a risk-stratified, evidence-based, and patient-centered approach to menopausal therapy.

In menopausal women necessitating menopausal hormone therapy, the existence of specific risk conditions and concomitant medication markedly affects the selection, timing of commencement, and length of treatment (Table 1).

### 1.1. Cardiovascular and Thrombotic Risk

Large contemporary observational studies have largely allayed long-standing worries over the elevated risk of venous thromboembolism (VTE) and ischemic heart disease (IHD) linked to oral MHT. Comprehensive population-based statistics indicate that the cardiovascular and thromboembolic risks vary among different kinds of menopausal hormone therapy and are significantly affected by the route of delivery. Transdermal estrogen regimens are consistently linked to a significantly lower risk of VTE when compared to oral preparations, according to large observational analyses based in the United Kingdom. This difference in risk appears to be largely independent of estrogen dose or type of progestogen. Recent research indicates that combined oral menopausal hormone therapy and tibolone are linked to a heightened risk of ischemic heart disease and thromboembolic events, whereas transdermal regimens exhibit a more advantageous cardiovascular risk profile.

The statistics align with the viewpoints established by the North American Menopause Society and the European Menopause and Andropause Society, both of which underscore the significance of route selection to reduce risk. Nonetheless, they underscore a continual shortcoming in the guidelines: the absence of precise, route- and dose-specific risk classification, especially for women with heightened baseline cardiovascular risk. For at-risk populations, such as women with pre-existing cardiovascular disease, obesity, limited mobility, or other prothrombotic risk factors, emerging evidence advocates for transdermal estrogen as the preferred initial route for MHT, when indicated, underscoring the necessity for guideline recommendations that more accurately represent the risk disparities evident in real-world data [14,15,16,17].

### 1.2. Cognitive Function

Numerous extensive studies and systematic reviews have investigated the association between menopausal hormone therapy and the risk of developing all-cause dementia and Alzheimer’s disease. Findings from the Women’s Health Initiative (WHI) trials initially indicated that estrogen-based hormone therapy commenced after age 65 was linked to a higher risk of dementia. In contrast, its use at younger ages—approximately between 50 and 55—seemed to have a neutral or possibly protective impact on cognition, although results have varied across different studies [18,19].

### 1.3. Polypharmacy and Drug Interactions

Polypharmacy remains an insufficiently addressed domain in menopausal hormone therapy guidance despite its growing relevance in women aged ≥65 years. Observational and pharmacoepidemiologic studies in older populations consistently demonstrate that the use of five or more concomitant medications is associated with a substantially increased burden of clinically significant drug–drug interactions and adverse drug events, a risk that becomes particularly relevant with the introduction of systemically administered estrogens, which undergo extensive hepatic metabolism via cytochrome P450 (CYP3A4) pathways (Table 2). These interactions may lead to unpredictable estrogen exposure, altered efficacy of co-administered therapies—particularly anticoagulants and cardiovascular medications—and an increased risk of thromboembolic or bleeding complications. Consequently, expert bodies increasingly emphasize the importance of comprehensive, pharmacist-led medication review and interaction screening as part of individualized MRT decision-making in older women.

No contemporary randomized controlled trials, nor guidelines, have evaluated MHT dose adjustment in the context of renal or hepatic impairment. Regulatory documents and pharmacokinetic analyses indicate reduced estrogen clearance in the presence of moderate-to-severe hepatic dysfunction, supporting a cautious approach with consideration of dose reduction or avoidance of systemic therapy in this population. In contrast, data regarding renal impairment remain limited, and current international MHT guidelines do not specify estimated glomerular filtration rate (eGFR) or Child–Pugh score thresholds for treatment eligibility. This lack of explicit guidance represents a critical evidence gap and underscores the need for individualized risk stratification in women with impaired organ function and high comorbidity burden [4,20,21].

In the absence of established guidelines for renal and hepatic impairment, a prudent interim clinical strategy may involve the use of transdermal estrogen formulations, administration of the minimal effective dose, and multidisciplinary consultation for cases of moderate-to-severe hepatic dysfunction. In patients with compromised renal function, treatment decisions should largely consider the total cardiovascular and thrombotic risk profile rather than renal function alone, due to the limited direct information about pharmacokinetic changes in estrogens in chronic kidney disease.

**Table 2:** Drug–drug interactions * between systemic menopausal hormone therapy and commonly prescribed medications in older women.

Drug Class	Agents	Mechanism of Interaction	Key Risk
Oral anticoagulants	Warfarin	E2-related changes in hepatic synthesis of coagulation factors	INR variability, thrombotic or bleeding events
Direct oral anticoagulants	Apixaban, rivaroxaban	Interaction on hemostasis	Increased bleeding or thrombotic risk
Antiplatelet agents	Aspirin, clopidogrel	Effects on coagulation and vascular endothelium	Increased bleeding risk
Statins	Simvastatin, atorvastatin	Shared CYP3A4 metabolism	Altered plasma concentrations
Antihypertensives	Diuretics, calcium channel blockers	E2-related fluid retention	Reduced blood pressure control
Psychotropic drugs	SSRIs, benzodiazepines, antipsychotics	Central nervous system modulation	Sedation, delirium, falls
NSAIDs	Ibuprofen, naproxen	Effects on platelet function and GI mucosa	Gastrointestinal bleeding

* The interactions mentioned indicate possible pharmacokinetic or pharmacodynamic pathways derived from established pharmacological characteristics and observational evidence. Polypharmacy does not necessarily indicate clinical impairment but highlights the necessity for personalized assessment and oversight.

### 1.4. Non-Hormonal Therapies

In patients with multimorbidity, the initial risk of adverse events is elevated, complicating therapeutic decisions. The existence of established cardiovascular disease, prior venous thromboembolism, or active oncological disease typically contraindicates the initiation of systemic menopausal hormone therapy. In such instances, preference is accorded to non-hormonal therapy methods or localized estrogen formulations for addressing genitourinary syndrome of menopause.

In recent years, the utilization of non-hormonal therapies for managing vasomotor symptoms has increased, particularly among patients with contraindications to menopausal therapy. Selective serotonin and noradrenaline reuptake inhibitors (SSRIs and SNRIs), gabapentin, and the novel class of neurokinin-3 receptor antagonists have demonstrated effectiveness in alleviating vasomotor symptoms. These medicines constitute a significant alternative for high-risk patient populations, particularly women with elevated cardiovascular or thromboembolic risk. Nonetheless, in geriatric patients with multimorbidity and polypharmacy, the implementation of non-hormonal therapy presents more complications. The existence of numerous comorbidities and concurrent pharmacotherapy heightens the risk of drug interactions, cumulative side effects, and reduced tolerance, particularly with centrally acting medications. SSRIs and SNRIs may pose risks of hyponatremia, QT prolongation, heightened fall risk, and interactions with anticoagulants or antiarrhythmics, whilst gabapentin is linked to drowsiness and cognitive impairment in geriatric patients. Despite neurokinin-3 receptor antagonists providing a novel non-hormonal alternative with a distinct mechanism of action, evidence on their long-term safety in patients over 65 years old with considerable comorbidities and polypharmacy is still insufficient. Current clinical guidelines lack explicit advice for the selection, dose, and monitoring of non-hormonal therapy for this diverse population, necessitating personalized clinical judgment and heightened caution in their application [22,23].

## 2. Materials and Methods

This study critically examines and assesses current clinical guidelines for menopause and menopausal hormone therapy, focusing on identifying at-risk patient populations and the complexity in establishing appropriate treatment options for these individuals. This narrative review was conducted between September and December 2025. A narrative approach was selected as it facilitates a thorough, summarizing, and interpretive synthesis of various sources, including clinical guidelines, position statements, and aggregated information from population-based studies, A narrative review design was deliberately chosen to allow integrative synthesis of heterogeneous evidence from clinical guidelines, observational studies, and pharmacological data, which cannot be meaningfully combined using quantitative meta-analytic methods in clinically vulnerable and underrepresented menopausal populations. A literature review was performed utilizing PubMed, Scopus, and Google Scholar. Keyword combinations included “menopause,” “menopausal hormone therapy,” “MHT,” “clinical guidelines,” “polypharmacy,” “multimorbidity,” “older women,” and “special populations.” Relevant publication reference lists were manually examined to locate supplementary sources. Research was prioritized according to its relevance to clinically vulnerable populations, study methodology (including guidelines, meta-analyses, and extensive observational studies), and timeliness.

The analysis encompassed prominent international and national clinical guidelines and position papers, including those from the North American Menopause Society (NAMS), the European Menopause and Andropause Society (EMAS), the National Institute for Health and Care Excellence (NICE), the American College of Obstetricians and Gynecologists (ACOG), the Endocrine Society, as well as pertinent guidelines from the Royal College of Obstetricians and Gynaecologists (RCOG), British Menopause society (BMS), and British Gynaecological Cancer Society (BGCS) in the United Kingdom (UK).

Publications were eligible for inclusion if they (1) addressed menopausal hormone therapy or non-hormonal alternatives, (2) focused on women aged ≥60 years or on populations with multimorbidity, polypharmacy, organ dysfunction, or cancer history, and (3) were clinical guidelines, position statements, systematic reviews, meta-analyses, large observational studies, or registry-based analyses. Only articles published in English between 2010 and 2025 were considered. Former guidelines (e.g., ACOG 2014, Endocrine Society 2015) were incorporated as they constitute the latest official recommendations from these organizations and continue to be extensively referenced in contemporary clinical practice. Relevant article reference lists were meticulously examined to locate supplementary sources. This review, being narrative in form, does not incorporate rigorous quality score or risk-of-bias evaluation. Evidence was analyzed by topic synthesis, focusing on clinical applicability, safety issues, and relevance to practical use. The gathered data underwent thematic analysis, revealing principal themes about therapy suggestions, risk–benefit evaluation, contraindications, and the necessity for a personalized treatment strategy.

### 2.1. Methodological Considerations and Limitations of Narrative Synthesis

This review was purposefully designed as a narrative synthesis to facilitate the integration of heterogeneous sources, including clinical practice guidelines, large observational studies, registry-based analyses, and pharmacological evidence, which cannot be effectively aggregated in quantitative meta-analytic models for clinically vulnerable menopausal populations. The process of source identification adhered to a systematic, although non-comprehensive, methodology. Clinical guidelines were selected according to international significance, citation frequency in clinical practice, and recency of revisions. Observational studies and systematic reviews were chosen to enhance guideline recommendations, especially in areas where guidance for older women, multimorbidity, polypharmacy, organ dysfunction, or cancer survivorship was scarce or nonexistent. While formal assessments of bias and quality rating were not conducted, evidence triangulation was utilized by comparing findings from independent data sources, guideline positions, and real-world safety signals. This methodology sought to diminish selective focus on particular research and to improve clinical relevance. The possibility of selection bias cannot be entirely eliminated in narrative evaluations. Nonetheless, transparency in guideline selection, clear eligibility criteria, and topic synthesis were employed to address this limitation.

When we encountered conflicting evidence, the data were assessed, taking into account study design, demographic characteristics, and consistency across sources.

The vulnerability framework presented in this review should be seen as a heuristic conceptual model intended to integrate persistent dimensions of clinical complexity recognized in guidelines and observational research. It is not designed to serve as a validated clinical decision-making instrument or risk assessment method. Instead, it offers a systematic framework for clinicians to evaluate cumulative risk variables in the personalization of menopausal hormone therapy choices.

### 2.2. Guideline Identification

We included:

1. NAMS—the 2022 Hormone Therapy Position Statement of the North American Menopause Society;
2. EMAS—the 2022 Menopause, wellbeing and health: A care pathway;
3. NICE NG23—Menopause: identification and management;
4. ACOG—Practice Bulletin Management of Menopausal Symptoms;
5. Endocrine Society—Treatment of the Symptoms of the Menopause: Clinical Practice Guideline;
6. UK/RCOG-linked resources—RCOG public/professional menopause pages (2025), Green-top guidance hub, and recent joint British Menopause Society and British Gynaecological Cancer Society guidance on menopause after gynecological cancer.

Where a guideline was older than 2019 (ACOG 2014; Endocrine Society 2015), we still included it because it is the latest official society-level guidance; it is still cited by subsequent reviews and users in clinical practice continue to rely on it.

Data for each clinical guideline were synthesized using a structured framework encompassing critical areas such as the target population, specifically women aged over 65, first-line and alternative therapies, dosage modifications, drug interactions, polypharmacy, monitoring and safety protocols, strength of evidence, implementation challenges, and accessibility.

### 2.3. Search Transparency and Prioritization of Evidence

The literature search was performed from September to December 2025 utilizing PubMed, Scopus, and Google Scholar. Search strings amalgamated controlled vocabulary with free-text phrases, encompassing: (“menopause” OR “menopausal hormone therapy” OR “MHT”) AND (“polypharmacy” OR “multimorbidity” OR “older women” OR “frailty” OR “cancer survivors” OR “renal impairment” OR “hepatic impairment”).

Emphasis was placed on:

International clinical guidelines and position statements published or revised between 2010 and 2025; systematic reviews and meta-analyses assessing cardiovascular, thromboembolic, cognitive, and safety outcomes; extensive observational cohort or registry-based studies involving populations aged ≥60 years or women with multimorbidity or polypharmacy; and regulatory evaluations and phase III clinical trials of non-hormonal therapies for vasomotor symptoms.

Previous guideline publications (e.g., ACOG 2014; Endocrine Society 2015) were preserved as they constitute the most current official society-level recommendations and are still referenced in modern clinical reviews and practice tools.

Triangulation of evidence was conducted by pairing guideline views with observational safety signals and pharmacological plausibility. In instances of inconsistent results, additional interpretive emphasis was placed on extensive population-based research, systematic reviews, and meta-analyses.

Sources of gray literature, such as reports from regulatory agencies (FDA, EMA) and statements from professional societies, were examined to find current advancements in therapy alternatives, specifically with neurokinin-3 receptor antagonists.

## 3. Results

Do the guidelines define “vulnerable groups”?

Current menopausal guidelines target vulnerable patient populations in a non-standardized manner (Table 3). NICE and the European Menopause and Andropause Society explicitly acknowledge the clinical complexity of these patients, multimorbidity, and the necessity for personalized monitoring and treatment. Nonetheless, EMAS ultimately lacks comprehensive recommendations on dose reduction for individuals with multimorbidity and polypharmacy. Conversely, the North American Menopause Society, the American College of Obstetricians and Gynecologists, and the Endocrine Society all offer a risk assessment grounded in contraindications to therapy, age, or periods of general vulnerability. ACOG identifies risk categories (VTE, CVD, and breast cancer) yet fails to offer a cohesive taxonomy of susceptibility. Recent UK guidelines from the RCOG, the British Menopause Society, and the British Society of Gynaecological Cancers emphasize the implementation of comprehensive, subgroup-specific advice, revealing a deficiency in overall menopause guidelines [24].

In all reviewed guidelines, systemic combined menopausal hormone therapy is the primary treatment for bothersome vasomotor symptoms, assuming there are no absolute contraindications. The adaptation of first-line treatment to individual risk differs, with NAMS and EMAS advocating for transdermal estrogen in women at heightened risk of venous thromboembolism or cardiovascular issues, but providing minimal quantitative guidance on dose modification. The guidelines for women with a history of gynecological cancer are significantly more comprehensive, as recent UK recommendations from the British Menopause Society and the British Gynaecological Cancer Society offer condition- and time-specific pathways, unlike the compact general guidelines for menopause [24]. Recommendations for dose reduction in renal or hepatic impairment and for managing polypharmacy are limited across all documents. Continuous monitoring and regular evaluation are universally advocated, with EMAS providing the most systematic framework for follow-up. However, specific instructions for commencing or discontinuing treatment, along with directives for the conduct of especially elderly women, are predominantly absent.

Table 4 summarizes notable research findings and systematic reviews published from 2019 to 2025 that highlight the therapy of menopausal symptoms in clinically vulnerable groups. The findings encompass information from extensive observational studies, assessments of registries across several nations, randomized controlled trials, and reviews, predominantly involving European populations.

Research conducted by Vinogradova et al. and Johansson et al. consistently indicated a reduced risk of venous thromboembolism and ischemic heart disease associated with transdermal estrogen compared to oral combined hormone therapy, while tibolone usage correlated with an elevated risk of ischemic heart disease. The findings were noted in extensive, unselected cohorts and underscore variations in cardiovascular safety based on the delivery method. Pourhadi et al. conducted a national case–control study that investigated cognitive impairment, revealing a correlation between combined hormone therapy and an elevated risk of dementia, especially with prolonged exposure. This study presents data on outcomes related to age and duration that are not expressly covered in the majority of clinical guidelines [19,25,26,27,28,29,30].

To summarize and clarify the interpretation of the evidence, the main data are categorized according to study design and level of evidence in Table 5. Most conclusions are based on observational studies, not randomized clinical trials, which require a cautious approach.

Practical clinical checklist for individualized MHT assessment in vulnerable women.

Prior to the initiation or continuation of menopausal hormone therapy, clinicians might consider the following systematic evaluation:

Patient-related domains.

- Age and years since menopause;
- Presence of ≥2 chronic diseases;
- History of cardiovascular or thromboembolic disease;
- Prior hormone-sensitive malignancy;
- Renal or hepatic impairment;
- Frailty or fall risk.

Medication-related domains.

- ≥5 concomitant medications;
- Use of anticoagulants or antiplatelets;
- CYP3A4 interacting drugs;
- Centrally acting medicines that increase fall risk.

Therapy-related considerations.

- Consider the transdermal route when cardiometabolic or thrombotic risk is elevated;
- Consider lowest effective dose;
- Consider non-hormonal alternatives when contraindications present;
- Perform medication reconciliation prior to initiation;
- Reassess benefit–risk balance periodically (6–12 months).

This checklist is intended to support structured clinical judgment rather than define eligibility criteria.

## 4. Discussion

This narrative study underscores a discrepancy between the advancing clinical practices in menopausal therapy and the directives outlined in established worldwide guidelines. Documents evaluated rarely prioritize vulnerable patient groups, despite the growing inclusion of women with multimorbidity, polypharmacy, organ impairment, advanced age, and cancer survivorship in menopausal care. Guideline development in complex populations necessarily relies on triangulation of observational data, pharmacology, and real-world safety signals, as randomized trials are unlikely to be feasible or ethical in many vulnerable subgroups.

The found discrepancies among guidelines indicate that vulnerability in menopausal treatment is primarily perceived as a collection of contraindications rather than a multifaceted clinical condition. This disparity is especially significant in older women, whose baseline risk is not only elevated but also more variable due to multimorbidity, functional decline, and drug burden.

The guidelines for dose adjustment, interaction algorithms, and monitoring methods stratified by age are inadequately specified. Through an analysis of the guidelines’ content and a comparison with current population-based studies and meta-analyses, we suggest that the evidence regarding differential risk based on MHT administration route, initiation age, and medication burden has advanced more rapidly than the guidelines’ implementation.

Menopausal hormone therapy is considered the most effective approach for managing unpleasant vasomotor symptoms in carefully selected patients, and treatment should be tailored through collaborative decision-making. Current evidence offers a more definitive understanding of vulnerable patient categories than most guidelines. Consideration should be given to age, comorbidities, and polypharmacy, which should not be regarded as contraindications to therapy, but rather as possibilities to tailor treatment through various MHT administration methods. Extensive population studies and meta-analyses consistently demonstrate route-dependent variations in cardiovascular and thrombotic risks among comorbid and elderly patients, with findings consistently suggesting that transdermal estrogen is linked to a more advantageous thromboembolic profile compared to oral therapy [14,15,16]. Comparable findings have been acquired from the research conducted by Johansson et al., which suggests an elevated cardiometabolic risk in specific individuals subjected to oral combination menopausal hormone therapy. There is no well-defined and established concept for suggesting transdermal estrogen as first-line therapy in women at risk or those with comorbidities and polypharmacy, despite the fact that NAMS and EMAS advocate it preferentially in people at higher risk.

Recent studies indicate that the effect of hormone therapy on cognitive performance is contingent upon the woman’s age and the length of treatment. A comprehensive Danish study [19] identified a correlation between combined hormone therapy and an elevated risk of dementia, particularly when the medication is prolonged or initiated at an older age; however, it did not establish a direct causal relationship. These statistics underscore the necessity for enhanced patient education and explicit measures for discontinuing medication in older women. Clinicians are currently left to make difficult judgments without particular direction since official risk assessment models, which mostly focus on cardiovascular disease and breast cancer, do not adequately encompass cognitive hazards.

**Figure 1:**
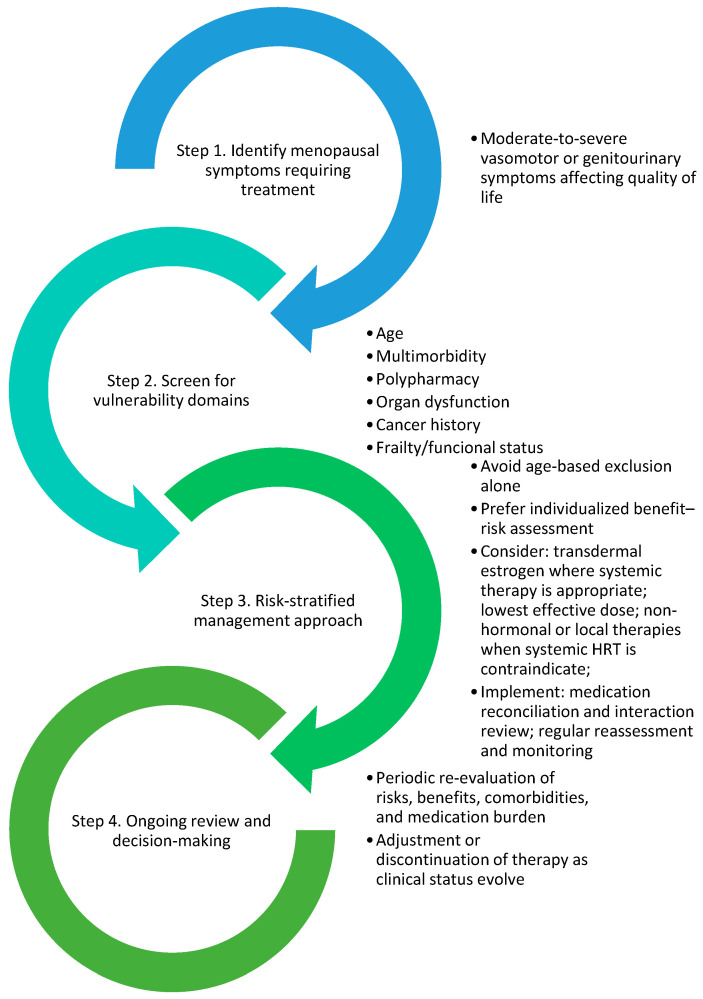
Author-proposed conceptual framework for identifying clinical vulnerability in menopausal hormone therapy. Vulnerability is characterized as a multifaceted construct that includes age, timing of therapy initiation, multimorbidity, polypharmacy, organ dysfunction, cancer history, and frailty or functional state. This framework serves as a conceptual and heuristic model and should not be construed as a validated clinical decision-making instrument or risk stratification algorithm.

A significant finding is that the majority of guidelines perceive vulnerability primarily as a collection of contraindications (such as prior VTE, established atherosclerotic disease, and active malignancy) rather than as a multifaceted construct that includes frailty, multimorbidity, organ dysfunction, functional status, and polypharmacy. This method is insufficient for older populations, as baseline risk is not only elevated but also more variable. Figure 1 illustrates a structured, flow-based framework synthesizing these vulnerability domains to support individualized menopausal hormone therapy decision-making.

Identical chronological age may be associated with significantly varied illness states, comorbidity profiles, and medication exposures. The UK guidelines for gynecological cancer survival indicate that more comprehensive, subgroup-specific therapy approaches and recommendations, tailored to the condition, time of commencement, and duration of exposure, are viable. The basic instructions for managing menopause and the advice for cancer patients differ in practice, indicating that prioritization—rather than the incapacity to develop a strategy—is the primary obstacle. Table 6 summarizes the key domains of vulnerability identified across the reviewed guidelines and supporting observational evidence, providing a structured framework for individualized menopausal hormone therapy decision-making. The domains presented in Table 5 are derived from recurring themes identified across the reviewed guidelines and supporting observational evidence, rather than representing novel risk thresholds.

Our view is that menopause recommendations should implement a unified approach for evaluating vulnerable patient populations using MHT, similar to the methods employed by oncology and cardiology to stratify patients based on frailty, risk scores, and treatment tolerance. Criteria for vulnerability must encompass: age categories (e.g., <60; 60–69; ≥70; ≥80), duration since menopause, frailty/functional status, significant comorbidities (cardiometabolic disease, chronic kidney disease, chronic liver disease, previous cancer, and thrombophilia), and criteria for polypharmacy (e.g., ≥5 medications). In the absence of such a taxonomy, the assertions in the recommendations are conceptually accurate yet practically insufficient.

A significant issue frequently undervalued is polypharmacy, defined as the concurrent use of numerous drugs. This is not merely a patient feature, but a significant factor that elevates the likelihood of adverse drug events, interaction cascades, and functional impairment. However, guideline recommendations generally assign the management of contacts to other entities (e.g., pharmacies) or exclude it altogether. The absence of this information is becoming increasingly concerning, as postmenopausal patients aged 65 and older often utilize anticoagulants/antiplatelet agents, antihypertensives, lipid-lowering therapies, antidiabetics, and psychotropics—drug classes that possess considerable potential for pharmacokinetic and pharmacodynamic interactions. Observational pharmacoepidemiological studies indicate a markedly heightened incidence of interactions in the context of polypharmacy, underscoring the necessity for thorough medication review as a fundamental component of menopausal hormone therapy decision-making (G. Rodriguez et al., 2022) [26]. The authors advocate for menopause guidelines to explicitly endorse pharmacist-led medication reconciliation at the commencement of therapy and at regular intervals for reassessment in women taking five or more concomitant medications, incorporating a standardized checklist of high-risk interaction categories. Polypharmacy should be regarded not as a definitive contraindication to menopausal hormone therapy, but as a significant risk indicator that necessitates a systematic assessment of medications and enhanced monitoring rather than automatic disqualification from treatment.

Impaired organ function is a significant concern that has not been sufficiently considered in the decision to commence menopausal hormone therapy. It is crucial to recognize that although guidelines recognize the complexity, they seldom delineate thresholds of renal function (eGFR cutoffs) or classifications of hepatic impairment (e.g., Child–Pugh) for eligibility, dosage modification, or monitoring. This situation becomes progressively intolerable due to the existence of illness-specific clinical standards (e.g., EMAS guidelines for chronic kidney disease and menopausal health) and the acknowledged pharmacological implications of hepatic metabolism of oral estrogen. The absence of stated thresholds compels clinicians to make arbitrary decisions, perhaps exacerbating unwarranted variability in practice.

Therapeutic alternatives have broadened, offering direction for the administration of hormonal drugs, particularly for patients for whom systemic hormone replacement therapy is contraindicated. Established nonhormonal alternatives (SSRIs, SNRIs, gabapentin, and fezolinetant) continue to be successful but present significant tolerability and interaction challenges in older persons and multimorbid populations [2,29]. In fact, guideline revisions should see nonhormonal therapy not as a supplementary appendix but as a primary approach for at-risk populations—integrated with polypharmacy and central nervous system safety considerations.

This review suggests revising recommendations in two ways simultaneously:

Application of risk stratification.

Algorithmic decision support should replace narrative risk discourse in guidelines. Paths should include transdermal vs. oral, progestogen, and non-hormonal options according to age, duration since menopause, baseline cardiometabolic and thrombotic risk, and cancer history.

Integrating comorbidity and medication burden into the basic model.

A feasible plan for the next decade should include structured drug reviews, deprescribing triggers, and organ function warnings. This is crucial since menopause care is transitioning from symptom control to quality of life for chronically ill women.

We believe that interdisciplinary guideline panels should include clinical pharmacology and geriatric medicine in order to address the difficulties of real-world clinical practice. This integration would also help to produce unambiguous drug dose modification tables, standardized monitoring schedules, and explicit discontinuation/continuation criteria for older age groups.

This analysis should be seen as a conceptual synthesis rather than a prescriptive clinical recommendation. The suggested framework, considering the narrative style and the diversity of accessible data, does not intend to set new treatment criteria or substitute existing evidence-based guidelines. It seeks to enhance systematic clinical reasoning in the management of menopausal symptoms in women with multimorbidity, polypharmacy, organ failure, or a history of malignancy. Future prospective research and guideline revisions are essential to authenticate organized methodologies for risk assessment and personalized decision-making in these populations.

### Strengths and Limitations

A principal strength of this review is its explicit focus on clinically vulnerable and underrepresented populations in menopausal care, including older women, those with multimorbidity, polypharmacy, organ dysfunction, and cancer survivorship. By prioritizing these groups, the review addresses a clinically important gap between guideline recommendations and real-world practice.

The real-world data presented in this review may be influenced by confounding factors, selection bias, and variability in study design, which must be taken into account when interpreting the results. The comparative analysis of major international and national menopause guidelines provides a structured overview of how vulnerability is defined, addressed, or overlooked across authoritative sources. Integrating guideline content with contemporary observational studies, registry data, and systematic reviews enhances clinical relevance and allows for the identification of discrepancies between emerging evidence and current recommendations. The narrative synthesis is well-suited to exploring clinical complexity and highlighting conceptual gaps, such as the absence of a unified taxonomy of vulnerability and practical prescribing algorithms.

## 5. Conclusions

In conclusion, current menopause recommendations are methodologically sound but may be operationally insufficient for susceptible categories that are increasingly represented in normal clinical practice. Evidence from large observational studies, meta-analyses, and increasing non-hormonal trial data supports a transition from implicit risk categories to explicit vulnerability frameworks that take into account age strata, comorbidity clusters, polypharmacy thresholds, and organ function. Updating guidelines to include practical algorithms and standardized monitoring will increase safety, eliminate unnecessary practice variance, and allow for fair access to symptom treatment across varied patient populations.

## Figures and Tables

**Table 1:** Major comorbidities, polypharmacy, and risks associated with systemic menopausal hormone therapy in women aged ≥65 years.

Comorbidity	Commonly Prescribed Medications	Clinical Implication	Key Risk
AH	ACE inhibitors, ARBs, diuretics, Ca-channel blockers	Fluid retention, increased blood pressure	Increased risk of stroke
IHD	β-blockers, statins, antiplatelet agents	Changes in lipid profile, prothrombotic effects	Increased coronary risk
VTE	Direct anticoagulants	Altered hemostatic balance	Increased risk of recurrent VTE
Type 2 DM	Metformin, SGLT2 inhibitors, GLP-1 insulin	Increased TG, IR	Increased CVR
CI	Antidepressants, antipsychotics	CNS effects of estrogens	Delirium, falls
Osteoporosis	Bisphosphonates, vitamin D	Additive skeletal effects	Reduced fracture risk

Abbreviations used: VTE—Venous thromboembolism; AH—arterial hypertension; IHD—ischemic heart disease; CNS—central nervous system; DM—type 2 diabetes mellitus; CVR—cardiovascular risk; CI—cognitive impairment; TG—triglycerides; IR—insulin resistance.

**Table 3:** Comparative overview of menopause guidelines (NAMS, EMAS, NICE NG23, ACOG, Endocrine Society, RCOG/BMS/BGCS) across vulnerability domains.

Guideline	Definition of Vulnerable Groups	First-Line Therapy	Alternatives	Dose Adjustment	Polypharmacy	Monitoring	Safety Framework
NAMS (2022)	Lists high-risk categories, but no formal definition	Individualized MHT; transdermal preferred in some risk contexts	SSRIs, SNRIs, gabapentin, and clonidine	Absent	Implicit, no structured DDI table	Periodic reassessment; no time interval	Strong emphasis on benefit–risk; no age-specific limits
EMAS (2022)	Explicit- mention of multimorbidity and frailty; still no operational criteria	MHT with risk stratification; transdermal for cardiometabolic risk	Non-hormonal if HRT contraindicated	Absent	Mentions need for pharmacist input; no algorithm	Reassessment at 6–12 months	Strong focus on cardiometabolic profiling
NICE NG23 (Updated 2024)	Explicit multimorbidity, complex cases	MHT first-line for VMS, if benefits outweigh risks	SSRIs/SNRIs, gabapentin	Absent	Absent	Reassessment at 3 months, then annually	Relevance in risk communication
ACOG (2014)	Implicit-only; focuses on contraindications	MHT for VMS; individualized	SSRIs, SNRIs, gabapentin	Absent	Absent	Non-specific monitoring	Emphasis on breast cancer/VTE risk
Endocrine Society (2015)	Implicit—high-risk groups	MHT for symptomatic women	SSRIs/SNRIs, gabapentin	Absent	Implicit	Annual review	Detailed risk discussion, but based on older evidence
RCOG/BMS/BGCS	Explicit for cancer survivors; strong oncologic focus	Non-MHT preferred for active cancer; MHT case-by-case	SSRIs/SNRIs, gabapentin, and fezolinetant	Absent	Implicit	Regular review; oncologic consultations	Strong cancer-risk framework

**Table 4:** Key recent studies and systematic reviews concerning vulnerable groups.

Citation	Design/Population	Therapy/Comparator	Main Outcome	Key Findings	Clinical Relevance for Vulnerable Groups
Vinogradova et al., (2019) [15]	Nested case–control (UK, >80,000 women)	Oral vs. transdermal MHT	Venous thromboembolism	Transdermal estrogen regimens had lower VTE risk.	Transdermal preferred in women with VTE or mobility risk.
Johansson et al., (2024) [25]	Nationwide cohort (Sweden)	Oral & transdermal MHT, tibolone	Ischemic heart disease, VTE	Oral combined—high risk for elevated IHD/VTE; tibolone—high IHD-risk.	Route of administration crucial for cardiometabolic risk.
Pourhadi et al., (2023) [19]	Case–control (Denmark, *n* ≈ 55,000)	Combined HRT vs. non-use	Dementia (all-cause, Alzheimer’s disease)	Combined HRT—increased dementia risk.	Age- and duration risk adjustment.
G. Rodriguez et al., (2022) [26]	Real-world registry (Europe)	Systemic MHT in polypharmacy	Adverse drug interactions	Polypharmacy doubled DDI probability.	Pharmacist review essential; need frail algorithms.
FDA & VA (2023) [27]	Regulatory review	Fezolinetant 45 mg daily	Liver function, VMS relief	Effective and safe; monitor liver function.	Non-hormonal option for hepatic or VTE risk.
Shapiro C. 2025 [28]	RCT (HT-unsuitable women, *n* = ~450)	Fezolinetant vs. placebo	VMS reduction, safety	Significant symptom improvement; good tolerability.	Expands options for cancer survivors or HT-contraindicated.
Young Moss [29]	Systematic review	SSRIs, SNRIs, gabapentin	Hot-flash control	Effective but high side-effect burden in the elderly.	Consider CNS and dementia risk in multimorbid women.

**Table 5:** Evidence strength and limitations for key clinical domains.

Domain	Type of Evidence	Key Findings	Main Limitations
VTE risk (route of administration)	Meta-analyses; observational studies	Transdermal estrogen—lower risk of VTE	Lack of RCT in high-risk populations
Cognitive outcomes	Observational (registry, case–control studies)	Increased dementia risk—late initiation and prolonged use of MHT	No causal inference; potential indication bias; heterogeneity across studies
Drug interactions	Pharmacoepidemiologic studies	Increased risk of drug–drug interactions in women taking ≥5 medications	Underreporting interactions; drug combination variability; unstandardized assessment
Cardiovascular risk	Observational studies meta-analyses	Route- and timing-dependent differences in cardiovascular outcomes	Limited RCT in older and multimorbid populations

**Table 6:** Clinical vulnerability checklist for individualized MHT assessment (conceptual tool).

Domain	Criteria Indicating Vulnerability *	Clinical Implications for MHT
Age and timing	Age ≥60–65 years; ≥10 years since menopause; late initiation of MHT	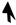 CV, VTE, and cognitive risk, lowest effective dose; avoid de novo systemic MHT ≥65 unless compelling indication
Multimorbidity	≥2 chronic conditions (CVD, DM, CKD, liver disease, cerebrovascular disease, neurocognitive disorders)	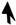 baseline risk; require individualized benefit–risk assessment; transdermal or non-hormonal options preferred
Polypharmacy	≥5 long-term medications—anticoagulants, antiplatelets, psychotropics, antiarrhythmics	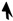 risk of drug–drug interactions and adverse events; pharmacist-led medication review recommended
Organ dysfunction	Reduced eGFR; hepatic impairment; drug-induced liver injury	Altered estrogen metabolism and clearance; consider dose reduction, transdermal route, or avoidance of systemic therapy
Cancer history	Prior breast or hormone-sensitive cancer; treated gynecological malignancy	Systemic MHTT contraindicated or restricted; case-by-case decision with oncology input; non-hormonal or local therapy favored
Frailty/functional status	Frailty, limited mobility CI, falls risk	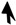 susceptibility to adverse effects; careful monitoring, simplified regimens, and reassessment are essential

* The vulnerability domains outlined in this table constitute a conceptual synthesis based on recurring themes from worldwide recommendations and corroborating observational evidence. They are not designed to serve as formal contraindications or evidence-based thresholds, but rather as a conceptual aid to support individualized clinical reasoning. The vulnerability paradigm presented in this review does not intend to establish new risk thresholds or supplant existing guideline-based contraindications. Instead, it represents a cohesive view of how vulnerability is implicitly considered in existing recommendations and observational evidence, although it lacks consistent operationalization. Due to the variability in aging and chronic disease progression, inflexible thresholds based on chronological age or individual circumstances may be clinically deceptive. A domain-based approach enables clinicians to consider cumulative risk while maintaining personalized decision-making.

## Data Availability

The original contributions presented in this study are included in the article. Further inquiries can be directed to the corresponding author.
